# The fatigue-induced alteration in postural control is larger in hypobaric than in normobaric hypoxia

**DOI:** 10.1038/s41598-019-57166-4

**Published:** 2020-01-16

**Authors:** Francis Degache, Émilie Serain, Sophie Roy, Raphael Faiss, Grégoire P. Millet

**Affiliations:** 1 Therapeutic and Performance Sports Institute, MotionLab, Le Mont Sur Lausanne, Lausanne, Switzerland; 2 School of Health Sciences, University of Applied Sciences and Arts Western Switzerland, Lausanne, Switzerland; 3 ISSUL Institute of Sport Sciences, Faculty of Biology and Medicine, University of Lausanne, Lausanne, Switzerland

**Keywords:** Neuromuscular junction, Neurophysiology

## Abstract

To test the hypothesis that postural control would be more affected by plantar flexors fatigue during acute exposure in hypobaric (HH) than in normobaric (NH) hypoxia or normobaric normoxia (NN). Twelve young male adults performed in a random order three experimental sessions (in HH and NH (F_i_O_2_ 0.139) at an altitude of 2950 m, and in NN at 500 m) composed of a bipedal postural control with eyes open on a posturographic platform before and after a plantar flexors fatiguing protocol. Center of pressure (C*o*P) trajectory and stabilogramm diffusion analyses (SDA) parameters were assessed. A two-way repeated measures analysis of variance was used to identify differences by examination of the group and time interaction. Surface of C*o*P trajectory analysis, increased at POST in HH (p < 0.001) and in NH (p < 0.01) compared to NN. SDA confirmed that PC was more altered in HH than in NH (p < 0.001) and NN (p < 0.05) at POST. The plantar flexor fatigue-induced alteration in postural control increased to a larger extent in HH than in NH or NN, suggesting an alleviating influence of the decreased barometric pressure *per se* and a mechanical influence of the higher breathing frequency in HH.

## Introduction

Postural control (PC) is a complex and multifactorial function requiring the ability to maintain the projection of the center of gravity in the base of support. Maintaining balance is a continuous and permanent re-establishment process^1,2^. The regulation of PC depends on sensorial information’s from visual, vestibular system located in the inner ear, proprioceptive and cutaneous receptor, an integration and treatment by the central nervous system, and finally an appropriate motor response. Under static or dynamic condition in several conditions measuring the center of pressure (C*o*P) displacement on a posturographic platform is one of the most used methods to analyses PC^1,3–5^. Another conceptual framework, called the stabilogram diffusion analysis (SDA), was applied to the analysis of the traditional statokinesigram for studying human PC^6,7^. In this approach, the C*o*P is modelled as fractional Brownian motion, which characterizes the effective stochastic activity of two PC systems during quiet standing: a short-term and long-term region. It has been suggested that short-term operates in an open loop mode and does not directly rely on sensory information, whereas the long-term region reflects closed loop control mechanisms that the human PC operates with sensory feedbacks. The transition between these two types of control is termed the critical point. This transition point was defining by the critical time intervals (Ct = Δ*t*_c_) and the critical mean square displacement (Cd = Δ*j*^2^) who quantify the spatial and temporal characteristics of the switching mechanisms.

Deterioration in PC due to muscular fatigue has been reported in several studies^1,8–13^. This decrease in the ability to maintain balance after a fatiguing task might be related to neuromuscular fatigue mechanisms that have both central and peripheral origins. This decline involves processes at all levels of motor pathways form the brain, including the central motor command (cortex, motoneurons), to the skeletal muscle, suggesting an impairment of the action potential transmission along the sarcolemma, the excitation-contraction coupling, the release and reuptake of calcium and the actin-myosin interaction^14,15^. Ankle plantar flexors (PF) are of particular interest because they play a major role in postural control during unipedal and bipedal quiet standing^13,16,17^. In this context, analyzing PF fatigue to demonstrate PC alterations seems relevant^1,9,12,17,18^.

Besides, an acute exposure to both hypobaric hypoxia (HH) and normobaric hypoxia (NH) leads to significant alteration in PC^2,4,19–21^. Recently, we suggested that PC was more altered in HH than in NH^4^ at rest. Hypoxic conditions can be defined as a combination of barometric pressure (PB) and an inspired fraction of oxygen (F_i_O_2_) that results in an inspired pressure of oxygen (P_i_O_2_ = F_i_O_2_ x PB-47) lower than a normoxic value of 150 mm Hg^22^. Indeed, hypoxia can be achieved by reducing PB (i.e. hypobaric hypoxia (HH) or by lowering the F_i_O_2_ while PB remains stable (i.e. normobaric hypoxia (NH)). It is well described that muscle fatigue alters postural control (1). Since hypobaric hypoxia is a more severe stimulus than normobaric hypoxia(4,23,24) and is known for accelerating the development of muscle fatigue^23^, one may speculate that postural control alteration would be greater in hypobaric hypoxia than in other environmental conditions.

To our knowledge, differences in PC have been reported only during acute passive exposure between HH and NH^4^. Since many popular sports (i.e., hiking climbing and mountain trail running) are practiced at altitude with reported effects on PC^19,20,24^, it is hence of interest to investigate the effects of a fatiguing exercise in HH vs. NH. Therefore, the main purpose of the present study was to investigate the effect of muscular fatigue in three different conditions (HH, NH, NN) on postural control in healthy subjects. Our hypothesis was that a muscular fatigue would alter more PC in HH than in NH or NN conditions.

## Materials and Methods

### Subjects

Twelve males without any history of neural disorder participated in this study (24 ± 3 years, 178.8 ± 7.0 cm, 78.3 ± 8.0 kg). All volunteers were low altitude residents (380–500 m) and had no significant altitude exposure at least one month prior the experiment. The local Medical Ethics Committee (CCVEM007/10, Sion, Switzerland) approved the study and written informed consent was obtained from each participant after explaining the experimental procedures and possible risks. Moreover, all experiments were performed in accordance with relevant guidelines and regulations described for research on human of local Medical Ethics Committee.

### Experimental design

The experimental design was conducted in three separate trials for twelves randomized subjects at matched conditions. NN measurements were performed in a well-ventilated laboratory outside the hypoxic chamber at an altitude of 500 m (F_i_O_2_ 20.9%, P_B_ 723 mmHg, Sion, Switzerland). The HH experiments were conducted at 2950 m (F_i_O_2_ 20.9%, P_B_ 541 mmHg) at Col de Gentianes (Valais, Switzerland). The NH sessions were realized in a hypoxic chamber (SL–400, ATS, Sydney, Australia), which is a well-ventilated 30-m^3^ room (2.4 m × 5.0 m × 2.5 m) with transparent glass panels. The hypoxic system allows sufficient hypoxic air input flow (up to 1000 l/min) in order to obtain a continuously stable F_i_O_2_ (altitude simulation by lowering the inspired fraction of O_2_). NH measurements were done at 500 m (Sion, Switzerland), at a “simulated” altitude of 2950 m (F_i_O_2_ 14.9%, P_B_ 723 mmHg). PB was measured at theses altitudes using a validated hand-held digital barometer (GPB 2300, Greisinger Electronic, Regenstauf, Germany; accuracy ±1.5 mmHg). F_i_O_2_ was measured using an oximeter (GOX 100 T, Greisinger Electronic) with an external sensor (GCO/GOO 370, Greisinger Electronic). PB and F_i_O_2_ were measured and recorded every four hours. We considered travel time to reach the location at 2950 m, travel time was of approximately 25 minutes by car and additionally 25 min by cable car. Travel times to the HH location were reproduced in the hypoxic chamber by decreasing F_i_O_2_ to the desired level progressively upon entering the hypoxic chamber.

P_i_O_2_ (103 vs 101 mmHg) and ambient temperature (24.4 vs 27.4 °C) were matched between for HH and NH, respectively).

#### Postural stability and fatigue protocol

Before fatigue protocol, subjects were prepared with a belt placed around the chest to calculate breath frequency (BF) (Bionomadix, Biopax System Inc, United States) and a finger pulse oximeter (WristOx® 3100, Nonin Medical, United States) was placed on the index of the right hand.

For all HH, NH, and NN conditions, a posturographic platform (Fusyo-Medicapteur, Toulouse, France; Dekra certification) was used to calculate the C*o*P trajectories. A computer with the Medicapteurs Winposture2000 software recorded data. The force plate measured 530 × 460 × 35 mm and was equipped with three pressure gauges (hysteresis < 0.2%). Signal processing was accomplished with a 16-bitA/D converter at 40 Hz^25^. Duration of test « Pre » and « Post» general session was 51.2 seconds, resulting in a 2048-point time series. Duration of each test between fatigue exercises was 25.6 s, allowing for 1024-point time series to be obtained to limit potential recovery time. Participants stood barefoot, double-leg on precise markers of the platform while trying to maintain postural stability during the trials in all sessions. Their legs were extended, and their feet formed a 30° angle relative to each other with an inter-malleolar distance of 5 cm.

The subjects were instructed to maintain their balance in the eyes open (EO) condition and to look at a fixed-level target at a distance of 0.9 m. The height of the visual target was adjusted for each subject. The verbal instructions were as follows:”Stand with your arms at your sides and look straight ahead while trying to maintain your postural stability to your best ability”. The intention of the instructions was to ask the participants not to fall and to limit their sway as much as possible.

**Figure 1:**
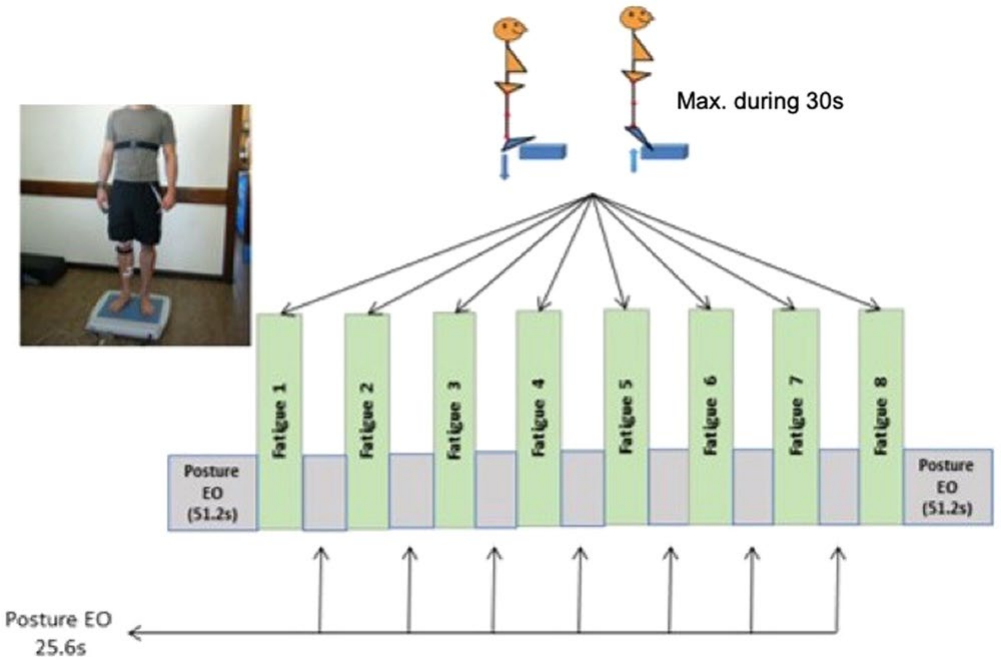
Testing Protocol. PC: postural control, EO: eyes open, MVC: maximal voluntary contraction.

The fatigue protocol started right after the initial posture measurement (Fig. 1). Subjects were standing on a step, and were asked to do “as much as possible plantar flexion keeping their knees straight” during 30 s. Number of repetitions, heart rate (HR, bpm), saturation (%), pain threshold (PT, mmHg) with a digital pressure algometer (FDX®, Wagner instrument, Greenwich, USA) were taken. The process was repeated 8 times. PC measurement after the fatiguing task is defined PC1, respectively until the last one, PC7. In parallel, subjective fatigue was evaluated by using the Borg visual analog scale (VAS) with a 100 mm horizontal line with “no fatigue” at 0 mm and “extremely fatigue” at 100 mm. At the end, postural measure (EO) followed by a MVC was done to finish the testing.

### Data acquisition

One standard postural parameter variable, the surface of C*o*P (90% confidence ellipse), was used to describe the subjects’ postural sway for each condition. In addition, SDA was performed for each subject, each recording time and each condition. The SDA summarize the mean square of C*o*P displacement as a function of the time interval between C*o*P comparisons^6^. This analysis was repeated for time intervals ranging from 1/40 to 10 s. The SDA parameters were extracted with Collins and Luca (1992)^6^ routine and the following parameters were taken for the medio-lateral (ML) and antero-posterior (AP): (1) The linear regression of the diffusion coefficients fort short- and long-term region (D*s*, D*l*)(mm^2^.s^−1^); (2) The point of intersection between the short- and long-term regions of the linear–linear plot is the critical point *C*^6^. The coordinates for the critical point (*Ct*, *Cd*) provide the measures of the *critical time interval*, i.e., Ct = Δ*t*_c_, and *critical value*, Cd = Δ*j*^2^.

All other variables, fatigue Borg scores (VAS, mm), pain threshold (PT, mmHg), saturation (%), HR (bpm), BF (Hz) were recorded. To ensure that subjects did the maximal PF during the fatiguing protocol, number of repetitions in the three conditions were taken into consideration.

### Statistical analyses

Mean ± standard deviation (SD) values were calculated for all variables of interest. After testing the assumption of normality of distribution (Kolmogorov-Smirnov test), the statistical tests were done with the software SigmaPlot (Version 11.0; systat software Inc., San Jose, CA). A a-posteriori sample size was performed.

A two-way repeated-measures analysis of variance (ANOVA) was used to identify differences by examination of the group (HH vs. NH vs. NN) x time (PRE, PC1, PC2, POST) interaction. Finally, a one-way ANOVA was done to analyse the difference in BF between the three conditions. Tukey post-hoc test was used to localize the differences between means. For all statistical analyses, a P value ≤ 0.05 was accepted as the level of significance.

## Results

### Summary statistics of postural control parameters

**Figure 2:**
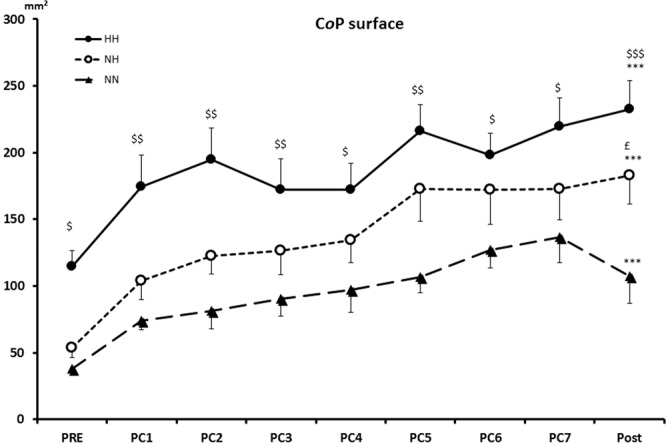
Postural control standards parameters, CoP surface (mm^2^). ^$^p < 0.05, $$p < 0.01, $$$p < 0.001 for difference between HH and NN. ^£^p < 0.05 for difference between NH and NN. ***p < 0.001 for difference with PRE.

*Standard parameters* of postural control are resumed in Fig. 2. *Co*P surface was higher in HH (p = 0.02) than in NN at PRE, and all along the fatiguing task until POST PC tests (p < 0.001). NH condition is statistically different from NN condition only at POST (p < 0.01). No statistical differences were found between HH and NH. For each condition, a significant time effect between PRE and POST was observed (p < 0.001).

**Figure 3:**
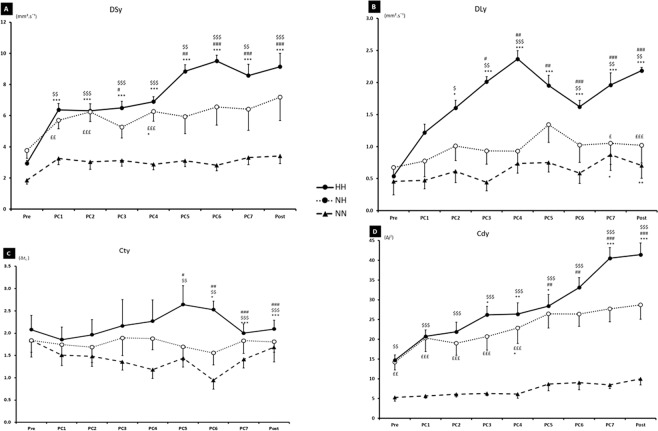
Results of SDA Parameters in the AP plan (y) with short term effective diffusion coefficient (D*sy*), long term effective diffusion coefficient (D*ly*), critical point (C*t*, C*d*) during the three conditions (HH: hypobaric hypoxia, NH: normobaric hypoxia, NN: normobaric normoxia). PC1, PC2…, correspond to the postural control measurement after the fatigue task. ^$^p < 0.05, ^$$^p < 0.01, ^$$$^p < 0.001 for difference between HH and NN. ^#^p < 0.05, ^##^p < 0.01, ^###^p < 0.001 for difference between HH and NH. ^£^p < 0.05, ^££^p < 0.01, ^£££^p < 0.001 for difference between NH and NN. *p < 0.05,**p < 0.01, ***p < 0.001 for difference with PRE condition.

*SDA* in the AP(y) plane is presented in Fig. 3. D*s*y (Panel A) HH was significantly higher than NH at PC3 (p < 0.05) and PC5 to POST (p < 0.001), and significantly higher (p < 0.01) compare to NN since PC1 to POST. In HH condition, D*s*y at POST were statistically different form PRE (p < 0.001). There was a statistically significant interaction between time and condition (p < 0.001, F = 4.937). D*l*y (Panel B), HH was again significantly higher than NH since PC3 to POST (p < 0.05) and with NN since PC2 to POST (p < 0.05). In HH, a significant time effect was observed from PC2 until POST (p < 0.001). There was a statistically significant interaction between time and condition (p < 0.001, F = 6.498). C*t*y (Panel C) showed only statistical differences from PC5 to POST between HH vs. NH (p < 0.05) and HH vs. NN (p < 0.01) conditions. Time effect was again observed in HH condition from PC6 to POST (p < 0.05). There was a statistically significant interaction between time and condition (p < 0.001, F = 3.174). C*d*y (Panel D) of HH and NH showed higher value than NN already in PRE condition (p < 0.01). In this panel D, a time interaction was observed in HH condition from PC3 (p < 0.05). There was a statistically significant interaction between time and condition (p < 0.001, F = 4.033).

**Figure 4:**
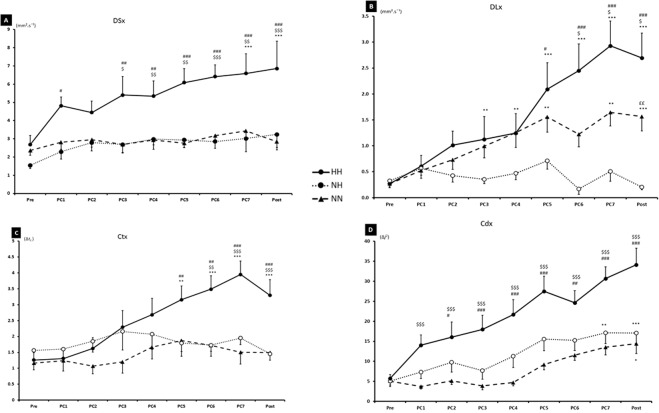
Results of SDA Parameters in the ML plan (x) with short term effective diffusion coefficient (Dsy), long term effective diffusion coefficient (Dly), critical point (Ct, Cd) during the three conditions (HH: hypobaric hypoxia, NH: normobaric hypoxia, NN: normobaric normoxia). PC1, PC2…, correspond to the postural control measurement after the fatigue task. ^$^p < 0.05, ^$$^p < 0.01, ^$$$^p < 0.001 for difference between HH and NN. ^#^p < 0.05, ^##^p < 0.01, ^###^p < 0.001 for difference between HH and NH. ^££^p < 0.01 for difference between NH and NN. **p < 0.05, ***p < 0.001 for difference with PRE condition.

*SDA* in the ML(x) plane showed similar results (Fig. 4). D*s*x (Panel A) and D*l*x (Panel B) showed statistical difference for HH vs. NH (p < 0.01) and HH vs. NN (p < 0.05) conditions, with higher value for HH. PRE to POST time effect was significant for D*sx* in HH condition (p < 0.001) and for D*l*x in HH and NN condition (p < 0.001). There was a statistically significant interaction between time and condition for both parameters (D*l*x, p < 0.001, F = 4.860 and D*s*x, p < 0.001, F = 2.852). C*t*x (Panel C) was higher in HH vs. NH since PC5 to POST (p < 0.01) and for HH vs. NN since P6 to POST. PRE to POST PC measurements increase more in HH condition (p < 0.001). There was a statistically significant interaction between time and condition (p < 0.001, F = 3.283). C*d*x (Panel D) was significantly higher in HH vs. NN from PC1 to POST (p < 0.001) and from PC2 to POST for HH vs. NH (p < 0.05). There was a statistically significant interaction between time and condition (p < 0.001, F = 2.676).

### Others variables

Number of repetitions over 30 s varied from 66 to 69 in HH, 64 to 67 in NH and 69 to 73 in NN. No time effect was found but statistical differences for the first three fatiguing periods were observed between NH and NN or between NH and HH (p < 0.05).

Fatigue Borg scores (VAS) and PT are presented in Table 1. Fatigue VAS increased and PT decreased at POST when compared to PRE (p < 0.001), excepted for the PT in NN condition but to the same extent in all conditions since no interaction was found.

**Table 1:** Borg visual analog scale (VAS) and pain threshold (PT) between the three different conditions (HH: hypobaric hypoxia, NH: normobaric hypoxia, NN: normobaric normoxia).

Conditions	Fatigue VAS		PT	
	*Pre*	*Post*	*Pre*	*Post*
HH	0.15 ± 0.48	5.77 ± 1.8***	66.71 ± 29.40	46.53 ± 18.74***
NH	0.17 ± 0.47	6.49 ± 2.01***	60.19 ± 21.35	48.36 ± 17.89***
NN	0.36 ± 0.90	5.03 ± 1.87***	54.35 ± 19.84	47.24 ± 14.52

***p < 0.001 for difference with PRE condition.

### Cardio-ventilatory responses

For all three conditions, HR increase and was significantly higher at POST compare to PRE without interaction between the conditions HH, NH and NN (Table 2). Inversely for SpO_2_ no time effect was found, but a significant difference (lower values) for HH vs. NN (p < 0.001) and NH vs. NN (p < 0.001) were observed at PRE and POST. BF in HH was significantly higher to NH and NN (p < 0.001) only at PRE.

**Table 2:** Heart rate (HR), saturation (SpO_2_). and breathing frequency (BF) between the three conditions (HH: hypobaric hypoxia, NH: normobaric hypoxia, NN: normobaric normoxia).

Conditions	HR (bpm)		SpO_2_ (%)		BF (Hz)	
	*Pre*	*Post*	*Pre*	*Post*	*Pre*	*Post*
HH	82.91 ± 15.08	110.89 ± 16.09***	90.09 ± 3.42^$$$^	91.22 ± 2.91^$$$^	0.33 ± 0.09^$$$###^	0.33 ± 0.08
NH	78.33 ± 11.76	118.89 ± 21.77***	89.00 ± 2.22^$$$^	90.11 ± 2.71^$$$^	0.22 ± 0.09	0.30 ± 0.08
NN	72.92 ± 10.18	120.11 ± 15.07***	96.25 ± 1.66	96.11 ± 2.15	0.23 ± 0.08	0.32 ± 0.10

***p < 0.001 for difference with PRE condition.

^$$$^p < 0.001 for difference with NN.

^###^p < 0.001 for difference with NH.

## Discussion

The main novel finding of this study is a plantar flexors fatigue induced significantly more postural instability when performed during exposure in HH than in NH. We also demonstrated that such muscle fatigue altered more PC in hypoxia (either in HH or in NH) than in normoxic condition. In the present study, we recorded that BF was higher in HH than in NH in PRE only. In several studies^26–29^, slight physiological responses between acute HH and NH have been reported concerning differences in cardio-ventilatory responses at rest. BF and HR are in general higher in brief exposure in HH but reach similar value in NH after 5 to 30 minutes following the onset hypoxia^26^. Hyperventilation represents a significant input for the PC regulation^30^ because it is correlated with the velocity of C*o*P displacement^31^, and a significant elevation of the surface of C*o*P^32^. It was suggested that immediate effects of hyperventilation led to a respiratory alkalosis, which induce an increase of nervous fibre’s excitability and a distortion of the proprioceptors^31,32^. Another explanation of PC impairments was showed by Hodges *et al*. (2002) suggesting that PC was directly related to an increase of the frequency and the amplitude of the respiratory movements^33^. One cannot rule out a direct mechanical influence of the higher breathing frequency in HH at least at the beginning of the protocol whereas other mechanisms (alkalosis, direct impact of lower PB on the vestibular activity) are likely inducing the larger PC alteration under increasing PF fatigue.

### Differences between HH and NH

Standards parameters, like C*o*P surface, were not significantly different in HH vs. NH. However, SDA values in both AP and ML planes that at the end of the fatiguing task were significantly higher in HH than in NH condition. This difference between HH and NH occurred generally after the third to the fifth fatiguing periods. Interpretation of SDA may offer more insight into the nature of the process controlling the C*o*P trajectories. Our results suggest that the stochastic activity of short- and long-term region were higher in HH condition, and that subjects took longer time to stabilise their body at POST in both planes (AP and ML) during first 10 seconds without taking into consideration somatosensorial afferences (corresponding to open-loop activity). An increase in short term stochastic activity could be due to increased co-activation of the ankle muscles^24^. Over the long-term region, the increase stochastic activity suggested that the actions taken by the PC were more frequent. This long-term change associated with muscular fatigue could be represented by two interpretations. The first one is a stiffening strategy, by increasing the activation of the antagonist muscles, because fatigue increase the frequency of action needed to regulate bipedal stance. The second one, peripheral and central level of the nervous system due to the fatigued muscle impact also the force production, which suggest that the motor output is more affected by the fatigue than the sensory system^12^.

The larger alteration of PC in HH condition could also be due to fatigue affecting proprioceptive feedbacks, which would be in link with the increase in critical point. These changes in the temporal interaction are supported by observation of increased reflex time, reduces proprioception and weaker muscle strength^24^.

It is known that postural stability is significantly deteriorated at high altitude in healthy subjects^5^ and that these alterations where significantly higher in HH than NH condition in the AP plane^2,4^. It was also suggested that postural instability was more influenced by change in PB than by change in fraction of oxygen^4^. It is known that hypoxia affects not only somatosensory information but also its integration and hypoxia inhibits the tonic vibration reflex in humans^34^. Moreover, activation of muscular mechanoreceptors (group I efferents) by tendon vibration is largely attenuated in acute and chronic hypoxemia in different animal species^35,36^.

In the present study, there were no significant correlations between cardiorespiratory changes and modifications of postural control. Since breathing pattern is different between NH and HH^37^, further research is required to investigate the effects of the thoracic cage movements on postural control at different altitudes in these two hypoxic conditions.

Despite that the present results confirm most of the existing literature (i.e., HH is a more severe stimulus than NH), the fact that the HH measurements were not performed in a hypobaric chamber but in “terrestrial altitude” and thus in a different location than in NN or NH, one cannot rule out that the conditions were not perfectly similar and may have slightly influenced the results. We assume that these differences were negligible.

Of interest was that a single-blinded protocol was applied between the NN and NH conditions; so that the subjects in the hypoxic chamber were not informed of the F_i_O_2_. However, for obvious reasons, blinding was not possible in the HH session. Therefore, one cannot exclude a placebo–nocebo effect on postural control.

### Differences between hypoxia conditions (HH and NH) and NN

Significant differences in C*o*P surface and SDA were observed between HH and NN conditions. All SDA parameters in HH, AP and ML, were more altered at 2950 m vs. 500 m. This is in line with previous findings who consistently reported that PC is impaired in an altitude-dependent manner^21^. Nordhal *et al*. (1998), demonstrated that vision is one of the first senses altered by the lower oxygen availability in acute hypoxia^2^. Considering the PC regulation mechanism, if vision is altered with acute hypoxia, higher disturbance on PC is observed.

Another explanation, could be in relation with the results of the study of Hoshikawa and al. (2000) who demonstrate that acute HH was related to an increase of the electromyographic activity of plantar flexors^19^, confirming that the sensory organization and motor output were influenced by acute HH exposure. Thus, the hypoxic stimulus seemed to have impacts on muscle activity^38^.

### The effect of fatigue protocol

Significant increase in C*o*P surface at POST in the three conditions were observed. The main finding of SDA was that for HH condition and in both plane, AP and ML, a significant interaction of time at POST were observed for almost all variables. Our fatigue protocol induce a moderate to heavy score on the fatigue Borg score at the end the fatiguing task. One study used the same method to evaluate the fatigue protocol and showed that a Borg score around 5.36 ± 2.11 impact PC in the AP measure^13^. The impact of muscular fatigue induces a greater contribution of the myostatic loops, the reflex response augment to increase stiffness joint and reduce postural sway^8^. Effect of fatigue also affects the descending drive, corresponding to a decrease of the efficacy of the cortico-spinal output. This decrease influences the motor neurons and affects the control of movement^39^. However, in several studies^1,8,10,11^, maximal isometric voluntary contraction was used as an index of fatigue to quantify the strength loss. This is one of the limitations of our study.

## Conclusion

In conclusion, the present study demonstrated that acute exposure to real altitude (HH, 2950 m) combined to a fatigue protocol affected PC to a larger extent than the same protocol at a simulated altitude and near sea-level in our specific population of young and active students. It remains unknown if similar findings would be observed in elderly and the consequences on the fall risks. The increase of CoP surface and SDA results showed that PC was altered in both AP and ML plane in HH condition. The present results suggest that postural control was firstly more influenced by change in barometric pressure and secondly by the fatigue protocol in hypoxic condition, that affect more the sensory pathway and the motor output. These observations have practical implications in all sport activities where PC is important and performed in altitude. It may be for safety reasons and prevent fall risks in trail running, climbing or mountaineering. It could be for training purpose in biathlon since shooting performance is deteriorated by altitude^40^.

### Limitations

Despite that the present results confirm most of the existing literature (i.e., HH is a more severe stimulus than NH), the fact that the HH measurements were not performed in a hypobaric chamber but in “terrestrial altitude” and thus in a different location than in NN or NH, one cannot rule out that the conditions were not perfectly similar and may have slightly influenced the results. We assume that these differences were negligible.

However, for obvious reasons, blinding was not possible in the HH session. Therefore, one cannot exclude a placebo–nocebo effect on postural control.
